# In vitro investigation of silica nanoparticle uptake into human endothelial cells under physiological cyclic stretch

**DOI:** 10.1186/s12989-014-0068-y

**Published:** 2014-12-24

**Authors:** Christian Freese, Daniel Schreiner, Laura Anspach, Christoph Bantz, Michael Maskos, Ronald E Unger, C James Kirkpatrick

**Affiliations:** REPAIR-lab, Institute of Pathology, University Medical Center of the Johannes Gutenberg University Mainz, European Institute of Excellence on Tissue Engineering and Regenerative Medicine, Mainz, Germany; Fraunhofer ICT-IMM, Mainz, Germany

**Keywords:** Cyclic strain, Hemodynamic conditions, HUVEC, Flex, Hemodynamic stress, Endocytosis and exocytosis of nanoparticles

## Abstract

**Background:**

In general the prediction of the toxicity and therapeutic efficacy of engineered nanoparticles in humans is initially determined using *in vitro* static cell culture assays. However, such test systems may not be sufficient for testing nanoparticles intended for intravenous application. Once injected, these nanoparticles are caught up in the blood stream *in vivo* and are therefore in continuous movement. Physical forces such as shear stress and cyclic stretch caused by the pulsatile blood flow are known to change the phenotype of endothelial cells which line the luminal side of the vasculature and thus may be able to affect cell-nanoparticle interactions.

**Methods:**

In this study we investigated the uptake of amorphous silica nanoparticles in primary endothelial cells (HUVEC) cultured under physiological cyclic stretch conditions (1 Hz, 5% stretch) and compared this to cells in a standard static cell culture system. The toxicity of varying concentrations was assessed using cell viability and cytotoxicity studies. Nanoparticles were also characterized for the induction of an inflammatory response. Changes to cell morphology was evaluated in cells by examining actin and PECAM staining patterns and the amounts of nanoparticles taken up under the different culture conditions by evaluation of intracellular fluorescence. The expression profile of 26 stress-related was determined by microarray analysis.

**Results:**

The results show that cytotoxicity to endothelial cells caused by silica nanoparticles is not significantly altered under stretch compared to static culture conditions. Nevertheless, cells cultured under stretch internalize fewer nanoparticles. The data indicate that the decrease of nanoparticle content in stretched cells was not due to the induction of cell stress, inflammation processes or an enhanced exocytosis but rather a result of decreased endocytosis.

**Conclusions:**

In conclusion, this study shows that while the toxic impact of silica nanoparticles is not altered by stretch this dynamic model demonstrates altered cellular uptake of nanoparticles under physiologically relevant *in vitro* cell culture models. In particular for the development of nanoparticles for biomedical applications such improved *in vitro* cell culture models may play a pivotal role in the reduction of animal experiments and development costs.

**Electronic supplementary material:**

The online version of this article (doi:10.1186/s12989-014-0068-y) contains supplementary material, which is available to authorized users.

## Background

In recent years the use of nanoparticles has become of interest in different scientific applications, such as medicine (drug delivery, diagnostics) [1,2], biomaterial science [3] or cell/tumor biology [4-6]. Thus, not only detailed physico-chemical characterization of nanomaterials is essential but also the assessment of the potential nanotoxicological impact on animals and humans. Due to the high number of newly synthesized materials and the requirement for rapid and convenient high-throughput screening of nanoparticle-cell interactions, *in vitro* cell experiments are used to evaluate the effects of nanoparticulate material on organisms. For a more detailed investigation of nanomaterials regarding their fate within organs, cells, or even cellular organelles, as well as transport properties through biological barriers (e.g., air-blood, or blood–brain barrier) more complex cell models have been developed [7-11]. These co- or triple-culture model systems consist of different cell types that exhibit a more physiological phenotype as a result of cell-cell interactions. These model systems are closer to the *in vivo* situation and thus more relevant for detailed investigation of nanoparticle-cell interactions *in vitro* especially when primary cells are used [12]. Although using such primary cell culture model systems is highly recommended they cannot completely mimic the *in vivo* situation. In particular, cells which are under permanent dynamic conditions, such as muscle cells, epithelial cells of the lung, vascular smooth muscle cells or endothelial cells making up blood vessels should be examined and analyzed in *in vitro* model systems that mimic the interactions of cells with nanoparticles under more physiological conditions. Endothelial cells that line the luminal side of the vasculature are exposed to hemodynamic forces such as cyclic strain and shear stress, caused by blood pressure and blood flow [13-16]. Since these mechanical stimuli have been identified as central modulators of vascular cell morphology and function, many studies have been published which describe the cellular processes regulating cell proliferation, apoptosis, differentiation, morphology, migration and secretory function [13,17]. Most of these studies focus on pathophysiological conditions and *in vitro* models have been set up to study, for example, atherosclerosis or intimal hyperplasia ([18], reviewed by [17]).

On account of the importance of *in vivo*-like experimental conditions and the reproducibility of cell culture experiments, the focus of the present study is the use of a stretch system to investigate the interaction of silica nanoparticles with primary human endothelial cells mimicking physiological conditions of the blood vessel. We used amorphous silica nanoparticles (aSNP; sicastar-redF) as model nanoparticles with different sizes (30 nm and 70 nm), but also investigated the impact of different surface modifications (−COOH, −NH2; or -OH) on cytotoxicity and uptake behavior of cells under various culture conditions. Although aSNPs are used in food additive and cosmetics, several studies have shown that aSNPs may have toxic effects on cells depending on concentration, morphology or size [19-21]. These nanoparticles can also have an effect on cells, which are not in direct contact with the nanoparticles but are influenced through a paracrine pathway [8]. In the investigation presented here we evaluate if more physiological conditions in the form of biomechanical stress compared to static conditions will change the results of nanoparticle toxicology assessment and the interaction of nanoparticles with cells. Moreover, the question will be addressed of whether changes need to be made to adapt standard *in vitro* experiments to more physiological models to achieve a more precise prediction of NP uptake *in vivo* using *in vitro* experiments.

## Results

### Particle characterization

Sicastar-redF nanoparticles with different sizes and various surface modifications were used as model nanoparticles in this study. We determined the sizes of the various amorphous silica nanoparticles (aSNPs) in different media by DLS. The data in Table 1 show that for the particles with a nominal size of 70 nm and regardless of their surface modification no significant changes in size occurred even after prolonged incubation times of 24 hours. In contrast, the 30 nm particles tended to agglomerate with time. Nevertheless, even under the high salinity conditions of the cell culture medium the overall colloidal stability remained similar and no macroscopic precipitation occurred.

**Table 1:** **Determination of sicastar-redF nanoparticle sizes in water and cell culture medium at different time points**

**Diluent**	**Time**	**30 plain**		**70 plain**		**70-COOH**		**70-NH**_**2**_	
		**D**_**h**_ **/nm**	**SD (%)**	**D**_**h**_ **/nm**	**SD (%)**	**D**_**h**_ **/nm**	**SD (%)**	**D**_**h**_ **/nm**	**SD (%)**
H_2_O	0 h	29.6	24	63.6	22	62.6	23	65.5	26
medium		63.7	59	63.5	13	61.0	11	62.8	10
H_2_O	24 h	33.0	14	72.2	22	64.1	10	66.0	10
medium		116.0	69	73.6	20	64.8	10	68.6	11

### Impact of aSNPs on cell viability and secretion of proinflammatory cytokines

After the physico-chemical characterization of the aSNPs the potential toxicity on primary human umbilical vein endothelial cells (HUVEC) was determined by using cell viability and cytotoxicity assays. The EC_50_ and LD_50_ values for HUVEC treated with various concentrations of aSNPs ranging from 0 to 6000 μg/ml on plastic cell culture dishes was determined (Additional file 1). The EC_50_ and LD_50_ values are summarized in Additional file 1 C. Based on this, concentrations of aSNPs that were not-toxic were used in all further studies. In addition, we demonstrated that aSNPs were free of endotoxin using an assay system described by Unger et al. [22], which is as sensitive as the commonly used Limulus Amebocyte Lysate (LAL) assay. It enables the detection of upregulated E-selectin expression on the surface of activated endothelial cells even if the nanoparticle suspension contains traces of LPS (see Additional file 2) [22]. Finally, HUVEC were seeded on flexible membranes, were grown for 48 hours under either stretch (1 Hz, 5% cyclic elongation) or static conditions before being treated with aSNPs for a further 24 hours (30 nm: 60 μg/ml or 70 nm: 150 μg/ml). The impact of aSNPs on endothelial cells was determined under two different culture conditions (static and stretch).

**Figure 1:**
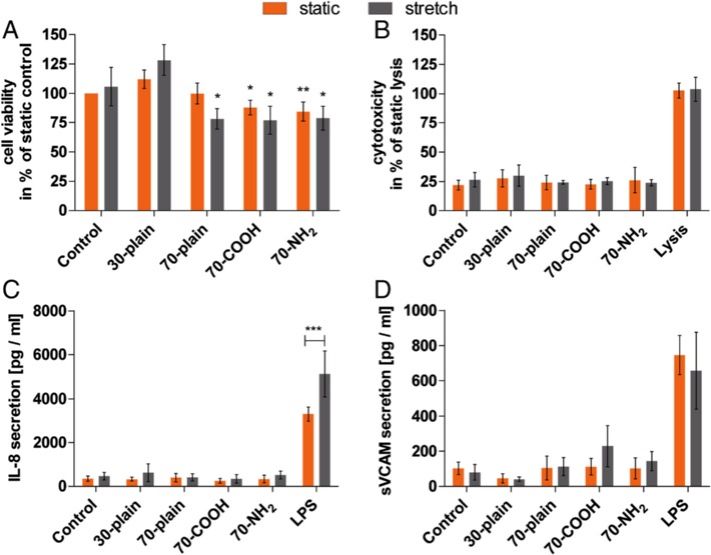
**Impact of stretch and nanoparticle treatment on cell viability and cytotoxicity and the expression of pro-inflammatory mediators.** HUVEC were seeded on flexible membranes and treated with sicastar-redF 30 nm-plain (60 μg/ml) or various 70 nm (150 μg/ml) sicastar-redF nanoparticles for 24 hours. **(A)** The MTS-Assay was used to determine cell viability and the acquired data were normalized to the untreated static control. **(B)** Cytotoxicity was determined by LDH-assay and data were normalized to static lysis. **(C)** Secretion of Interleukin- (IL-) 8 was investigated by ELISA. LPS-stimulated cells were used as positive control. **(D)** Soluble vascular cell adhesion molecule (sVCAM) was determined by ELISA, while the secretion of sVCAM after LPS treatment was used as positive control. Results shown are means ± SD calculated using the results of at least three independent experiments. For cell viability: *: P <0.05, **: P <0.01 compared to the appropriate untreated control (ONEway ANOVA with Dunnetts *t*-test); for IL-8 secretion: ***: P <0.001 (TWOway ANOVA with Bonferroni post-test).

In Figure 1A the cell viability of HUVEC treated with aSNPs under static or stretch conditions is shown. Cell viability was measured by the bioreduction of tetrazolium compound into a formazan product (MTS-assay) in metabolically active cells. The results demonstrate that stretch compared to static conditions did not affect cell viability of HUVEC regardless of whether the cells have been additionally treated or not with 30 nm or 70 nm aSNPs for 24 hours (Figure 1A). Nevertheless, compared to the appropriate stretched and untreated control cell viability of HUVEC, which have been stretched and then treated with 70 nm particles was significantly decreased up to 77% (±12%). The same results were observed for cells which have been treated with surface-modified 70 nm particles under static conditions (70 - COOH: 88% ±6%; 70 - NH_2_: 84% ±8%). In contrast the 70 nm - plain nanoparticles did not significantly affect the cell viability under static conditions compared to the untreated control. In addition, 30 nm particles did not decrease cell viability regardless of whether cells have been stretched or cultured under static conditions compared to the respective untreated controls. In addition to the MTS assay cell toxicity was also determined after treatment with the various nanoparticles by measuring the release of lactate dehydrogenase into the culture medium from cells with a damaged membrane (Figure 1B). It was found that neither the 30 nm nor the 70 nm aSNPs caused any cytotoxic effects, even in combination with stretch compared to the static untreated cells. In addition to the determination of cytotoxic effects, the protein expression of pro-inflammatory mediators (Interleukin-8 (IL-8), soluble vascular cell adhesion molecule (sVCAM)) was investigated using ELISA. The results shown in Figure 1C, D demonstrate that aSNPs in the concentrations used did not increase the secretion of the chemokine IL-8 in HUVEC (Figure 1C). The various conditions under which HUVEC were cultured and treated did not affect the secretion of IL-8, even when cells were incubated with various aSNPs in parallel. Furthermore, the secretion of sVCAM during stretch conditions and nanoparticle treatment was not enhanced compared to the untreated control (Figure 1D). A moderate but not significant increase in the secretion of sVCAM was observed under stretch conditions and the simultaneous treatment of HUVEC with 70-COOH silica particles. In addition to the data shown in Figure 1C, D the secretion of interleukin-6 (IL-6) and soluble intercellular cell adhesion molecule (sICAM) has been investigated (data not shown). The data obtained confirmed that endothelial cells under different culture conditions and after the treatment with various aSNPs did not show any pro-inflammatory response compared to the untreated cells.

### Internalization of aSNPs into HUVEC under different culture conditions

**Figure 2:**
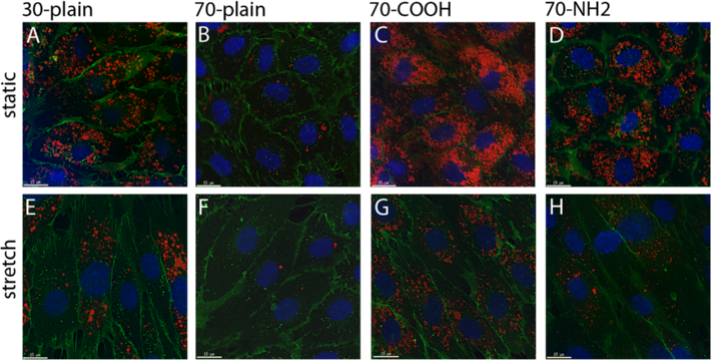
**Internalization of silica nanoparticles into HUVEC under static and stretch culture conditions.** HUVEC were seeded on flexible membranes and grown to confluence under different culture conditions (static or stretch conditions). Afterwards cells were treated with silica nanoparticles (red) under static **(A–D)** or stretch **(E–H)** conditions for 24 hours. Cells were washed and afterwards fixed with paraformaldehyde. Cell nuclei were stained with Hoechst dye (blue) and the membrane associated CD31 with the appropriate primary and secondary antibodies (green). Fluorescent microscopy was performed with a DeltaVision-microscope and a with the same exposure times. Scale bar: 15 μm.

In order to determine if effects were observed on the morphology of endothelial cells microscopic images were obtained after treatment with the nanoparticles under the different culture conditions (Figure 2). The representative images shown in Figure 2 demonstrate that the morphology of HUVEC is more elongated when cultured under physiological stretch conditions while the static cultured endothelial cells exhibit a morphology which is mostly referred to as a ‘cobblestone’-like morphology. Even though cells are elongated under stretched conditions, they are well connected to the neighbouring cells and express the cell adhesion molecule CD31 at the cell periphery similar to cells cultured under static conditions. However, more aSNPs are internalized under static conditions compared to the cells grown under dynamic stretch condition. 30 nm-plain aSNPs are internalized in a higher amount compared to the 70 nm-plain aSNPs and nanoparticles with a carboxylated surface are preferentially internalized by endothelial cells compared to 70 nm-plain or –NH2 aSNP.

### Internalization of aSNP into HUVEC under static, stretch and mixed culture conditions

**Figure 3:**
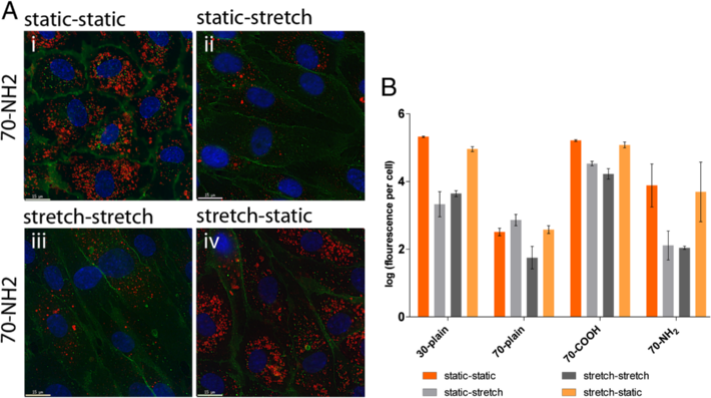
**Image analysis of internalized silica nanoparticles into HUVEC under static, stretch and mixed culture conditions. (A)** HUVEC cultivated on flexible membranes under static (i + ii) and stretch (iii + iv) conditions were treated with silica nanoparticles under static (i + iv) or stretch (ii + iii) conditions for 24 hours. Cells were extensively washed, fixed and stained (CD31 (green)). Cell nuclei were counterstained with Hoechst dye (blue). Scale bar: 15 μm. **(B)** Images were used to quantify the cell number and the fluorescent signal coming from internalized silica nanoparticles within the cells using Keyence analyzing software (4 images each). Results shown are means ± SD.

The functionality status of endothelial cells, static versus stretched and the conditions of exposure to the nanoparticles were also examined. In this case, cells that had been cultivated under stretch or static conditions were treated with NPs under these conditions or transferred to the other condition (static - static, static - stretch, stretch - static, stretch - stretch). In Figure 3A the internalization of NH2-modified silica NP is shown. In Figure 3A i and A iii the results of the basic conditions are presented which have already been described above (Figure 2). Under static treatment conditions more particles were internalized by the cells compared to the stretch conditions. The quantification of the fluorescent signals within the cells confirmed these observations (Figure 3 B; orange and dark grey bars). Taken together and based on the culture-treatment conditions (Figure 3A (ii + iv)) and the analysis in Figure 3B it is apparent that the treatment condition under which the cells were incubated with nanoparticles is relevant and influences the amount of internalized nanoparticles. However, the results also show that the pre-cultivation of cells under different conditions also has an impact on the amount of internalized nanoparticles even though the differences in uptake under ‘static-static’ (orange) and ‘stretch-static’ (yellow) or ‘stretch-stretch’ (dark grey) and ‘static-stretch’ (grey) conditions is not so obvious (Figure 3A and B). Representative images of other nanoparticles internalized under the four different conditions are depicted in Additional file 3. The combined summary of the results indicates that the differences in the amount of nanoparticle uptake under stretch and static conditions is due to the conditions under which nanoparticles interact with cells (static or stretch). Nevertheless, the altered and more differentiated phenotype of HUVEC observed under stretch conditions also appears to play a pivotal role in the internalization process of silica nanoparticles into HUVEC.

### Investigation of the mechanism of altered nanoparticle uptake under stretch culture conditions

**Figure 4:**
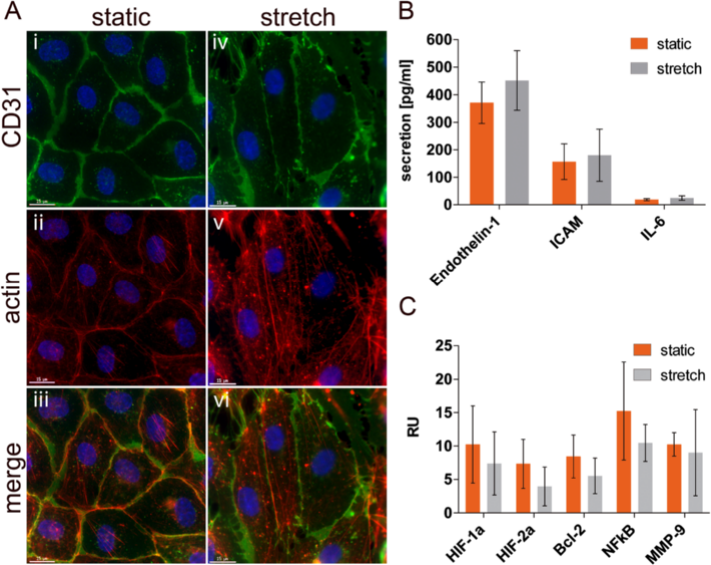
**Stretch-induced changes in morphology and expression of proteins related to cell stress and exocytotic events. (A)** HUVEC cultivated on flexible membranes under static (i – iii) and stretch (iv – vi) conditions were stained for CD31 (green) and actin (red). Cell nuclei were counterstained with Hoechst dye (blue). Scale bar: 15 μm. **(B)** Quantification of secreted growth factors or inflammatory mediators under both culture conditions detected by ELISA. **(C)** Comparison of the protein expression levels of cell stress- and angiogenesis-related proteins of stretched and unstretched cells have been investigated by protein array. Results shown are means ± SD.

The mechanisms which could lead to an altered uptake behavior under physiological *in vitro* conditions were investigated. First, cell stress caused by cyclic strain as a potential modulator of endocytosis was analyzed. The expression profile of 26 cell stress-related proteins of stretched and unstretched cells has been investigated at protein level. Selected protein levels are shown in Figure 4C. None of the proteins examined showed an altered expression in HUVEC grown under stretch conditions compared to static culture conditions. Neither apoptotic-related proteins (Bcl-2) nor transcription factors NFƙB and hypoxia-induced factors (HIFs) were induced under the physiological culture conditions. In addition to the unchanged expression of the transcription factor HIF, the protein levels of related downstream metabolites which are involved in extracellular cellular matrix metabolism such as MMP-9 remained unaltered. In Figure 4A the actin filaments of the cells cultured under various conditions are shown. In Figure 4A (i - iii) it can be seen that actin is located at the cell borders and is co-localized with the membrane protein, CD31 (green, resulting in a yellow staining). Under stretch conditions (Figure 4A (iv – vi)) the actin fibers are located within the cell and are less co-localized with CD31. This additional change in the cell morphology (more elongated cells after stretch) shows that the stretch protocol was effective. Since it has been shown that changes in the structure of the cytoskeleton can impact membrane traffic and it has been reported that stretch increases the tension of the plasma membrane thereby subsequently impacting endocytotic and exocytotic properties of cells [23-26], then there should be different amounts of internalized nanoparticles in cells as a result of an altered endocytosis or exocytosis rate under static and stretch culture conditions. Since mechanical stimuli such as stretch are known to stimulate the secretion of several markers (endothelin-1, tissue-type plasminogen activator, cytokines) into the cell culture medium [27-30], we specifically analyzed the secretion of endothelin-1 (ET-1), a relevant factor which is released under stretch conditions by ELISA [27]. Figure 4B shows that the secretion of ET-1 under the different conditions was not significantly induced. Also the secretion of interleukin-6 (IL-6) and soluble ICAM (sICAM) was not increased under stretch conditions in culture.

**Figure 5:**
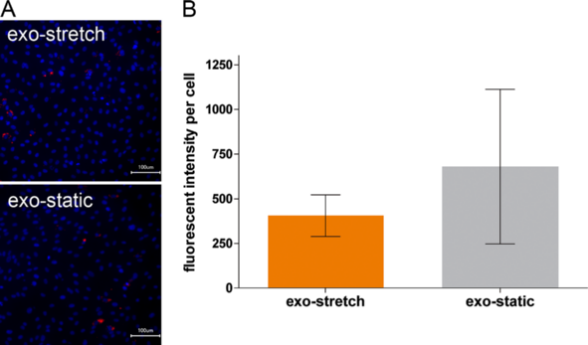
**Uptake and quantification of exocytosed 70 nm-COOH silica nanoparticles using a cell assay system.** HUVEC seeded on flexible membranes were treated for 24 hours with aSNPs under static conditions. NP suspension was removed, cells extensively washed, incubated with fresh medium under flex or static condition for 24 hours. The medium including the exocytosed aSNPs was used to treat HUVEC grown onto 96 well plates for further 24 hours. Afterwards these cells were washed, fixed, and analyzed for internalized aSNPs by image analyses. **(A)** Representative images of cells that internalized exocytosed aSNPs. Cell nuclei counterstained with Hoechst dye (blue). Scale bar 100 μm. **(B)** Quantification of relative fluorescent intensity depicted as means of 24 images per condition.

To determine if the lower amount of aSNP within the cell under stretch conditions is a result of an increased exocytotic event and not due to decreased endocytosis, HUVEC were treated with aSNP under static conditions for 24 hours. Cells were extensively washed, fresh medium was added and afterwards cells were cultured under stretch or static conditions. After 24 hours of incubation the supernatant was transferred to HUVEC seeded onto 96 well plates for a further 24 hour period. Following this, stretched and static cells were fixed and analyzed for internalized aSNPs by fluorescence microscopy (data not shown). No differences in the amount of nanoparticles within the cells were observed. The cells on the 96 well plates, which were incubated with the supernatants of the static and stretch plates were analyzed for the presence of internalized silica nanoparticles. In the control cells incubated with medium no red fluorescence could be detected. For the 70-plain and 70-NH2 aSNPs only a slight amount of NPs could be found within the cells. Thus, the quantification was focused on 70-COOH nanoparticles (Figure 5). In Figure 5A the images of the nanoparticle uptake by HUVEC are presented. A number of images were quantified by image analysis of the red signal. The data indicate that the uptake properties into the cells did not significantly differ regardless of whether the cells were incubated with the supernatant of cells, which have been stretched or cultured under static conditions (Figure 5B). Thus, by measuring the secretion of ET-1 or the internalization rate of exocytosed aSNPs it appears that the decreased amount of aSNPs within the cells under stretch conditions was not due to increased exocytotic events.

## Discussion

Nanoparticles as drug delivery systems or imaging tools may be useful in improving biomedical therapies. The accreditation of nanoparticles as biomedical tools usually starts with *in vitro* investigations. The development of *in vitro* models which mimic the *in vivo* situation is highly desirable and may lead to the reduction of animal experimentation which could subsequently reduce the development costs for pharmaceutics. Static cell culture is used for the determination of cell reactions, such as toxicity or the formation of reactive oxygen species. However, endothelial cells are permanently exposed to hemodynamic forces such as shear stress and cyclic stretch [15]. Although the effect of shear stress has been investigated [31-34], the current study focusses on the effects of sterile, endotoxin-free sicastar-redF nanoparticles on endothelial cells under stretch culture conditions. Amorphous silica nanoparticles (aSNPs) of various sizes and surface modifications appear to affect endothelial cells differently under static and stretch cell culture conditions. It was also shown that cytotoxic effects were not altered, although the internalization of the aSNPs under various culture conditions differed.

The characterization of the NPs used was the first step in the present investigations. The data presented in Table 1 show that the characteristics of the nanoparticles diluted in the cell culture medium used are comparable to previously reported studies [35]. The presence of serum proteins gives rise to a slight agglomeration and mean particle diameters in the range of 100–200 nm were measured (data not shown). This behavior is in accordance with the expectations: The formation of a protein corona gives rise to a disturbance of the mechanism of colloidal stabilization of the particles (electrostatic stabilization) and results in the formation of agglomerates [36,37]. The determination of potential toxic effects of aSNPs under various culture conditions was also analyzed. Interestingly, the impact of the aSNPs on HUVEC was not significantly increased under stretch conditions compared to the static conditions and may be due to the application of a non-pathophysiological stretch (5% stretch, 1 Hz) [38-40]. The reduction of the metabolic activity of HUVEC after exposure to 70 nm-COOH or 70 nm-NH2 aSNPs may be due to the high internalization rate of these nanoparticles. However, no toxicity was detected after 24 hours of exposure (LDH assay). Regarding the 30 nm- and 70 nm-plain aSNPs, the results are in accordance with the data measured for endothelial cells (ISO-HAS-1) previously published by our group [35]. Furthermore, Nabeshi et al. also determined an impact of 30 nm and 70 nm sicastar-redF particles on the proliferation of HaCaT cells after 24 hours of incubation [41]. However, the toxic effect on cells under physiological stretch conditions was not investigated. In addition, the unaltered secretion of IL-8 and sVCAM by HUVEC under various culture conditions and simultaneous treatment with NPs show that neither the applied stretch nor the nanoparticles activate an inflammatory response in endothelial cells. Nevertheless, the cells that were treated with LPS under stretch show a significantly increased IL-8 secretion compared to the cells treated with LPS under static conditions, which might be due to the more physiological character of these cells under stretched conditions.

The main outcome of these studies was that under physiological stretch conditions less aSNPs were internalized by HUVEC compared to static cell culture conditions. To our knowledge, the correlation of cyclic stretch and internalization of nanoparticles into endothelial cells has not been reported previously. Rouse et al. examined the effects of quantum dots (QD) on keratinocytes under stretch conditions and found that the interaction of QD and keratinocytes (HEK) was increased under cyclic strain [42]. Unfortunately, the mechanisms behind the altered uptake behavior of QD into HEK were not further investigated. Many studies have reported the effects of stretch on the phenotype and protein expression profile of HUVEC, but these studies usually focused on hypertension (≥10%) [39] of endothelial cells which mimics a pathological condition in endothelial cells, e.g. arteriosclerosis [18] and not cells under normal conditions. In the present study the physiological elongation of 5% was investigated to determine if the differences in the uptake properties of aSNPs are due to a more physiological and differentiated cell phenotype, inflammatory responses and cell stress or are a result of a higher exocytosis rate of the cells grown and treated under stretch conditions. The results of the studies demonstrated that the treatment condition has greater impact on the interaction effects of the aSNPs on the cells than a more differentiated and stretch-adapted phenotype of the cells (comparison of the four culture-treatment conditions). In addition, a pre-incubation of cells under stretch has a small impact on the uptake rate of aSNPs into HUVEC. Therefore, it appears that the changed morphology and phenotype of the ECs prior to exposure to NPs plays a secondary role in the uptake of aSNP into HUVEC *in vitro*. An analysis of the most prominent stress mediators of ECs by a protein array demonstrated that cell stress factors were not mediators involved in influencing the uptake behavior of aSNPs under stretch conditions. Various studies have shown that cellular stress impacts the endocytotic rate of metabolites [43,44]. Since no enhanced cell stress factors under stretch conditions could be detected it appears that these factors are not responsible for the decreased endocytosis of the aSNPs. Furthermore, studies of HUVEC under stretch conditions indicated that stretching cells results in a decreased endocytosis and an increased exocytosis rate of the cells which apparently counteracts the membrane tension caused by stretch [45]. This increased exocytosis rate can be indirectly measured by an enhanced secretion of growth factors such as tissue plasminogen activator or endothelin-1 [27,46-49]. However, a comparison of the amount of the secretion of ET-1 under flex and static cell culture did not exhibit significant differences in the amount of ET-1 after applying 5% stretch. The discrepancy may be due to the amount of stretch applied or the time points which were chosen for the measurement of ET-1. To determine if aSNPs are exocytosed more efficiently under stretch conditions as a consequence of counteracting the membrane tension studies were undertaken to determine if more aSNPs are released under stretch conditions. This was found not to be the case and is contrary to the results of the uptake of exocytosed NPs in static culture of HUVEC (Figure 5). A net increase of exocytotic events could not be detected and thus the decrease in endocytotic events might be responsible for the results observed. The model described by Sinha et al. may explain the lower amount of endocytotic events [50]. The model defines that under certain conditions the stretch of membranes will be counteracted by the flattening of caveolae. Following the flattening process of the membrane by the disappearance of caveolae it is likely that a decreased endocytosis and increased exocytosis are counteracting the stretch and complete the initial response at longer time periods of stretch. Due to the physiological stretch applied flattening of the membrane with subsequent caveolae disappearance could be an explanation why the exocytosis rate in our studies, analyzed by ET-1 secretion and aSNP exocytosis, is not altered under the two different culture conditions. Thus, increased exocytosis is not responsible for the lower number of particles within the cells but membrane flattening could be the reason for less endocytotic events during stretch. However, previous studies have analyzed the cell entry mechanisms of aSNPs and no co-localization of aSNPs with caveolin-1 or clathrin-heavy chain was observed. However, localization with flotillins, apparently responsible for the uptake of NPs by caveolin- and clathrin-independent mechanisms were observed for NPs in endothelial and lung epithelial cells [9,35]. Moreover, the mechanism of flotillin-dependent uptake is similar to caveolae-dependent endocytosis [51], and therefore, membrane flattening might also affect the flotillin-controlled uptake mechanism.

Further studies also demonstrated that the arrangement of actin fibers differ in stretch cultured cells compared to static cultured cells. This demonstrates that cells are morphologically affected by the applied stretch and that the elongation by 5% leads to a slight alteration in the cytoskeleton arrangement and the cell morphology which is comparable to the situation *in vivo* [52,53]. The effects on the cytoskeleton caused by stretch also play a pivotal role in mechanotransduction [24,26,28,54]. Due to the strong attachment of the cytoskeleton and the plasma membrane, changes in the arrangement of the cytoskeleton also affect endocytotic events [45,55]. Han et al. showed that flow also affects the cytoskeletal arrangement and that this occurrence is related to an altered uptake of spherical polystyrene particles coated with PECAM antibodies [34]. Thus, the direct influence of stretch due to the membrane (flattening process) and the rearrangement of the cytoskeleton may lead to a changed uptake rate of aSNP in HUVEC in a concerted manner.

Other explanations are possible for the altered internalization of aSNPs observed in these studies under static and stretch conditions. Since the expression of factors which are involved in extracellular matrix (ECM) metabolism are also known to be induced by stretch, the interaction of nanoparticles and the cell membrane of HUVEC might be affected with the resulting consequence being an altered uptake rate of nanoparticles under stretch conditions. We have demonstrated that matrix metalloproteinases-9 expression is unaltered under stretch and also the expression of transcription factors (e.g. HIFs), which can induce the expression of MMPs, is not induced. The expression of MMP-2 and collagen type I and IV have been analyzed on protein level but no significant differences could be detected (data not shown). Nevertheless, by adapting *in vitro* experiments to more *in vivo*-like conditions a development of a more *in vivo*-like ECM and a polarization of cells may occur which may also change the interaction of NPs and cell membranes. The consequences of this could be an altered internalization rate of NPs into cells. In addition the sedimentation of the nanoparticles may be a prominent factor impacting the uptake processes and is dependent on the nanoparticle interaction with the membrane [56]. This might alter the uptake rate of aSNP into HUVEC under stretch, since the stretch conditions applied might lead to medium movement above the cells. However, this movement of NPs above the cells would mimic the movement of NPs in blood as it occurs *in vivo*.

The results of these *in vitro* studies demonstrate that *in vitro* test systems have to be adapted to more physiological conditions that mimic *in vivo* conditions more closely. These studies have also shown that differences occur with four highly similar but slightly modified aSNPs in their interactions with HUVEC under certain stretch conditions. Further studies, such as changing the frequency, time, elongation, the type of endothelial cells and testing different NPs will be necessary to give a more complete picture of how stretch affects the uptake of nanoparticles into endothelial cells. A device to investigate in parallel the impact of stretch and shear stress on nanoparticle uptake and transport across lung cells has been published by Huh et al. [57]. Such a 3D organ-on-a-chip model adapted to other organs would be a valuable addition to evaluating the impact of shear stress and stretch on nanoparticle uptake in unique cell types and may lead to reduction of animal studies.

## Conclusions

In conclusion the present study shows that in addition to shear stress, cyclic stretch also affect the interaction of nanoparticles and endothelial cells and such systems should be highly relevant for designing specific targeted nanoparticulate drug delivery strategies.

## Methods

### Particle characterization

Silica nanoparticles (aSNP; sicastar-redF) were purchased from micromod Partikeltechnologie GmbH, Rostock (Germany). All particles were fluorescently labelled (λ_ex_ =585 nm) and particle diameters were 30 nm and 70 nm. The larger particles were purchased not only with plain silica surface (Si–OH/Si–O^−^), but also with carboxy-(−COOH) and with amine-(−NH_2_) modified surface to examine the influence of surface properties. Particle sizes were determined by Dynamic Light Scattering (DLS), and thus, the reported sizes are z-weighted mean values of the hydrodynamic diameter. Particle diameters were measured in cell culture medium (Endothelial Cell Basal Medium (ECBM); PromoCell) and, for reference, in water (containing 2 mmol/L sodium bromide to guarantee optimum colloidal stability). Two time points were chosen (0 and 24 hours), representing the start and the end point of the cell experiment. DLS measurements were performed using a Microtrac NANO-flex instrument (with a 180° backscattering setup). The data analysis mode “Monodisperse” was used for the evaluation of the measurements. Further characteristics of the particles such as number of nanoparticle per milliliter or per milligram can be found on the manufacture’s homepage (www.micromod.de). Further data of the nanoparticle characteristics are summarized in Additional file 4.

### Cell isolation and culture

Umbilical cords were obtained from randomly selected healthy mothers. All procedures were in agreement with the ethical standards of the University Medical Center of the Johannes-Gutenberg University Mainz (§ 14 AVB, Abs. 3) and with the Helsinki Declaration. Primary human umbilical vein endothelial cells (HUVEC) were isolated according to a previously published method [58,59]. Cells were cultured in medium M199 (Sigma Aldrich), 20% fetal calf serum (Life Technologies), 2 mM Glutamax I (Life Technologies), 100 U/100 mg/ml penicillin/streptomycin, 25 mg/ml sodium heparin (Sigma-Aldrich) and 25 mg/ml endothelial cell growth factor supplement (ECGS, Becton Dickinson) on gelatin-coated cell culture flasks (greiner bio-one) upon isolation. After the first passage cells were cultured in ECBM, 15% fetal calf serum, 2.5 ng/mL basal fibroblast growth factor, 10 μg/mL sodium heparin (both Sigma-Aldrich) and 100 U/100 mg/ml penicillin/streptomycin (hereinafter referred to as ECBM culture medium) on gelatin-coated cell culture flasks. Cells were used for the experiments in passage 2–4 and are cultured under standard cell culture conditions (5% CO_2_, 95% humidity, 37°C).

### Assessment of cell viability, cytotoxicity and E-selectin expression

Cells were seeded onto fibronectin-coated 96-well plates in ECBM culture medium and cultured to confluence. Cells were exposed to various concentrations of aSNPs for 24 hours. The nanoparticles were diluted in ECBM, supplement mix (PromoCell) and 100 U/100 mg/ml penicillin/streptomycin (hereinafter referred to ECBM stimulation medium). Cell viability was measured using the CellTiter 96 AQueous non-radioactive assay (Promega) as recommended by the manufacturer. For the detection of cytotoxicity caused by the treatment of aSNPs, 50 μl of the cell supernatant was used to carry out the CytoTox 96 non-radioactive cytotoxicity assay (Promega). The lactate dehydrogenase (LDH) release after cell lysis (1% TritonX 100 (Sigma-Aldrich)) into the medium was used to determine 100% LDH release and the release of LDH of cells, which have been treated with the appropriate volume of nanoparticle diluent was used as control. Particle interference with the assay systems was not detected. After measuring cell viability cells were washed with phosphate-buffered saline and fixed with methanol/ethanol at room temperature for 15 minutes. The following E-selectin determination by cell adhesion molecule enzyme immunoassay (CAM-EIA) was performed as previously described to evaluate aSNPs for the initial screening for presence of endotoxin [22,60]. Cells treated with 1 μg/ml lipopolysaccharide (LPS) was used as positive control and set to 100% E-selectin expression. The determination of cell viability and cytotoxicity for cells cultured on flexible membranes were carried out as described above using cells which were cultured under stretch or static conditions and treated with 60 μg/ml or 150 μg/ml of 30 nm or 70 nm silica nanoparticles, respectively.

### Exposure to Nanoparticles during cyclic stretch

HUVEC were seeded onto fibronectin-coated flexible silicon membranes (BioFlex Culture Plate (FlexCell International Corporation)) with ECBM culture medium. 24 hours after seeding, cells were exposed to cyclic stretch for 48 hours (5% elongation (sinus) and a frequency of 1 Hz) using a FX-4000 Tension Plus FlexerCell strain unit and a FlexLink controller. This system was connected to a base plate holder, which was equipped with six round 25-mm loading posts and a vacuum pump. Control cells were also seeded on the membranes but were cultured under static conditions without any stretching for the same time period. Cells were treated with 60 μg/ml (6.37 × 10^12^ particles/well) or 150 μg/ml (1.26 × 10^12^ particles/well) of 30 nm or 70 nm aSNPs, respectively. We chose these concentrations to prevent an overload of the cells with nanoparticles and applying concentrations which have been demonstrated to be non-toxic. Nanoparticles were diluted in ECBM stimulation medium. For mixed conditions cells were treated for 48 hours under the first condition and 24 hours (incubation time for the nanoparticles) under the second condition.

### Enzyme- linked immunosorbent assay (ELISA)

After exposure to the nanoparticles under different culture conditions the supernatants of the cells were diluted in the appropriate diluent and analyzed via ELISA (DuoSet, R&D Systems) for secreted soluble pro-inflammatory mediators or growth factors (sVCAM, sICAM, IL-8, IL-6, and endothelin-1) as recommended by the manufacturer.

### Immunofluorescent staining, microscopy, and image quantification

After treatment with nanoparticles, membranes with HUVEC were washed repeatedly with HEPES buffer and PBS, fixed with 3.7% paraformaldehyde for 15 minutes and stained with specific antibodies. For staining of the cell membrane, mouse anti-human PECAM-1 (CD-31; Dako) was used. Nuclei were counterstained with Hoechst 33342 dye (Sigma-Aldrich). The membranes were embedded with GelMount (Biomeda, Natutec) and analyzed via fluorescence microscopy (Olympus IX71 with Delta Vision system (Applied Precision) or BZ9000 (Keyence)). To analyze the amount of internalized nanoparticles, images were taken with BZ9000 (20x objective) using the same magnification and exposure times. BZ Analyzer software (Keyence) was used to count cell nuclei and to determine the relative fluorescent intensity.

### Cellular stress and angiogenesis array

The expression profile of stretch and static cell culture of HUVECs was investigated for proteins related to cell stress or angiogenesis using the Human Cell Stress Array Kit or Human Angiogenesis Array Kit (both R&D Systems) according to methods recommended by the manufacturer. The protein concentration of each sample was determined using the BCA protein assay kit (Pierce).

### Data analysis

GraphPad Prism version 5.04 software (Prism) was used for data analysis.

## Additional files

Additional file 1:
**Determination of EC**_**50**_ **and LD**_**50**_ **of various aSNPs in Huvec.** (A) HUVEC were treated with various nanoparticles in a concentration range of 0 to 6000 μg/ml for 24 hours and EC_50_ was determined by MTS assay. Untreated cells were set to 100% cell viability (B) The supernatant of the cells was examined for the amount of released LDH to determine the LD_50_. Lysed cells were used as positive control and set to 100%. (C) Summary of EC_50_ and LD_50_ for the various aSNPs. (donors ≥3). Additional file 2:
**E-Selectin expression of Huvecs to determine endotoxin contamination of the nanoparticles.** HUVEC were grown on 96-well plates and treated with 60 μg/ml 30 nm-plain or 150 μg/ml 70 nm aSNPs for 4 hours. Cells were washed, fixed and E-selectin expression was determined by CAM-EIA as described by Unger et al. 2014 (see references). 1 μg/ml lipopolysaccharide was used as positive control and set to 100% E-selectin expression while untreated cells were used as negative control. (2 donors in triplicate). Additional file 3:
**Uptake of various aSNPs into HUVEC under static, stretch and mixed culture conditions.** HUVEC cultivated on flexible membranes under static (a + b) and stretch (c + d) conditions were treated with silica nanoparticles under static (a + d) or stretch (b + c) conditions for 24 hours. Cells were extensively washed, fixed and stained (CD31 (green)). Cell nuclei were counterstained with Hoechst dye (blue). Scale bar: 15 μm. Additional file 4:
**Nanoparticle characteristics provided by the manufacturer (** www.micromod.de **).**

## References

[1] Ittrich H, Peldschus K, Raabe N, Kaul M, Adam G (2013). Superparamagnetic iron oxide nanoparticles in biomedicine: applications and developments in diagnostics and therapy. Fortschr Röntgenstr.

[2] Kiriyama A, Iga K, Shibata N (2013). Availability of polymeric nanoparticles for specific enhanced and targeted drug delivery. Ther Deliv.

[3] Mohtaram NK, Montgomery A, Willerth SM (2013). Biomaterial-based drug delivery systems for the controlled release of neurotrophic factors. Biomed Mater.

[4] Heidel JD, Davis ME (2010). Clinical developments in nanotechnology for cancer therapy. Pharm Res.

[5] Sanna V, Sechi M (2012). Nanoparticle therapeutics for prostate cancer treatment. Maturitas.

[6] Sechi M, Sanna V, Pala N (2014). Targeted therapy using nanotechnology: focus on cancer. Int J Nanomed.

[7] Hermanns MI, Kasper J, Dubruel P, Pohl C, Uboldi C, Vermeersch V, Fuchs S, Unger RE, Kirkpatrick CJ (2010). An impaired alveolar-capillary barrier *in vitro*: effect of proinflammatory cytokines and consequences on nanocarrier interaction. J R Soc Interface.

[8] Kasper J, Hermanns MI, Bantz C, Maskos M, Stauber R, Pohl C, Unger RE, Kirkpatrick JC (2011). Inflammatory and cytotoxic responses of an alveolar-capillary coculture model to silica nanoparticles: comparison with conventional monocultures. Part Fibre Toxicol.

[9] Freese C, Unger RE, Deller RC, Gibson MI, Brochhausen C, Klok H, Kirkpatrick CJ (2013). Uptake of poly(2-hydroxypropylmethacrylamide)-coated gold nanoparticles in microvascular endothelial cells and transport across the blood–brain barrier. Biomater Sci.

[10] Hemmelmann M, Metz VV, Koynov K, Blank K, Postina R, Zentel R (2012). Amphiphilic HPMA–LMA copolymers increase the transport of Rhodamine 123 across a BBB model without harming its barrier integrity. J Control Release.

[11] Brandenberger C, Rothen-Rutishauser B, Mühlfeld C, Schmid O, Ferron GA, Maier KL, Gehr P, Lenz A (2010). Effects and uptake of gold nanoparticles deposited at the air–liquid interface of a human epithelial airway model. Toxicol Appl Pharmacol.

[12] Kunzmann A, Andersson B, Thurnherr T, Krug H, Scheynius A, Fadeel B (2011). Toxicology of engineered nanomaterials: focus on biocompatibility, biodistribution and biodegradation. Biochim Biophys Acta.

[13] Kakisis JD, Liapis CD, Sumpio BE (2004). Effects of cyclic strain on vascular cells. Endothelium.

[14] Pradhan S, Sumpio B (2004). Molecular and biological effects of hemodynamics on vascular cells. Front Biosci.

[15] Azuma N, Duzgun S, Ikeda M, Kito H, Akasaka N, Sasajima T, Sumpio BE (2000). Endothelial cell response to different mechanical forces. J Vasc Surg.

[16] Abe R, Yamashita N, Rochier A, Nixon A, Abe R, Madri JA, Sumpio BE (2011). Varying effects of hemodynamic forces on tissue factor RNA expression in human endothelial cells. J Surg Res.

[17] Anwar M, Shalhoub J, Lim C, Gohel M, Davies A (2012). The effect of pressure-induced mechanical stretch on vascular wall differential gene expression. J Vasc Res.

[18] Okada M, Matsumori A, Ono K, Furukawa Y, Shioi T, Iwasaki A, Matsushima K, Sasayama S (1998). Cyclic stretch upregulates production of interleukin-8 and monocyte chemotactic and activating factor/monocyte chemoattractant protein-1 in human endothelial cells. Arterioscler Thromb Vasc Biol.

[19] Jonghoon Choi QZVRNSWMESVMHPLG (2009). Comparison of cytotoxic and inflammatory responses of photoluminescent silicon nanoparticles with silicon micron-sized particles in RAW 264.7 macrophages. J Appl Toxicol.

[20] Huang X, Teng X, Chen D, Tang F, He J (2010). The effect of the shape of mesoporous silica nanoparticles on cellular uptake and cell function. Biomaterials.

[21] Lin W, Huang Y, Zhou X, Ma Y (2006). *In vitro* toxicity of silica nanoparticles in human lung cancer cells. Toxicol Appl Pharmacol.

[22] Unger RE, Peters K, Sartoris A, Freese C, Kirkpatrick CJ (2014). Human endothelial cell-based assay for endotoxin as sensitive as the conventional Limulus Amebocyte Lysate assay. Biomaterials.

[23] Apodaca G (2002). Modulation of membrane traffic by mechanical stimuli. Am J Physiol Ren Physiol.

[24] Morris CE, Homann U (2001). Cell surface area regulation and membrane tension. J Membr Biol.

[25] Abbott NJ, Dolman DEM, Drndarski S, Fredriksson SM (2012). An improved *in vitro* blood–brain barrier model: rat brain endothelial cells co-cultured with astrocytes. Methods Mol Biol.

[26] Hamill OP, Martinac B (2001). Molecular basis of mechanotransduction in living cells. Physiol Rev.

[27] Carosi JA, Eskin SG, McIntire LV (1992). Cyclical strain effects on production of vasoactive materials in cultured endothelial cells. J Cell Physiol.

[28] Wang N, Butler JP, Ingber DE (1993). Mechanotransduction across the cell surface and through the cytoskeleton. Science.

[29] Park JM, Borer JG, Freeman MR, Peters CA (1998). Stretch activates heparin-binding EGF-like growth factor expression in bladder smooth muscle cells. Am J Physiol.

[30] Barron V, Brougham C, Coghlan K, McLucas E, O’Mahoney D, Stenson-Cox C, McHugh PE (2007). The effect of physiological cyclic stretch on the cell morphology, cell orientation and protein expression of endothelial cells. J Mater Sci Mater Med.

[31] Bhowmick T, Berk E, Cui X, Muzykantov VR, Muro S (2012). Effect of flow on endothelial endocytosis of nanocarriers targeted to ICAM-1. J Control Release.

[32] Farokhzad OC, Khademhosseini A, Jon S, Hermmann A, Cheng J, Chin C, Kiselyuk A, Teply B, Eng G, Langer R (2005). Microfluidic system for studying the interaction of nanoparticles and microparticles with cells. Anal Chem.

[33] Kusunose J, Zhang H, Gagnon MKJ, Pan T, Simon SI, Ferrara KW (2013). Microfluidic system for facilitated quantification of nanoparticle accumulation to cells under laminar flow. Ann Biomed Eng.

[34] Han J, Zern BJ, Shuvaev VV, Davies PF, Muro S, Muzykantov V (2012). Acute and chronic shear stress differently regulate endothelial internalization of nanocarriers targeted to platelet-endothelial cell adhesion molecule-1. ACS Nano.

[35] Kasper J, Hermanns MI, Bantz C, Koshkina O, Lang T, Maskos M, Pohl C, Unger RE, Kirkpatrick CJ (2013). Interactions of silica nanoparticles with lung epithelial cells and the association to flotillins. Arch Toxicol.

[36] Tenzer S, Docter D, Rosfa S, Wlodarski A, Kuharev J, Rekik A, Knauer SK, Bantz C, Nawroth T, Bier C, Sirirattanapan J, Mann W, Treuel L, Zellner R, Maskos M, Schild H, Stauber RH (2011). Nanoparticle size is a critical physicochemical determinant of the human blood plasma corona: a comprehensive quantitative proteomic analysis. ACS Nano.

[37] Tenzer S, Docter D, Kuharev J, Musyanovych A, Fetz V, Hecht R, Schlenk F, Fischer D, Kiouptsi K, Reinhardt C, Landfester K, Schild H, Maskos M, Knauer SK, Stauber RH (2013). Rapid formation of plasma protein corona critically affects nanoparticle pathophysiology. Nat Nanotechnol.

[38] Oluwole BO, Du W, Mills I, Sumpio BE (1997). Gene regulation by mechanical forces. Endothelium.

[39] Maul TM, Chew DW, Nieponice A, Vorp DA (2011). Mechanical stimuli differentially control stem cell behavior: morphology, proliferation, and differentiation. Biomech Model Mechanobiol.

[40] Zheng W, Jiang B, Wang D, Zhang W, Wang Z, Jiang X (2012). A microfluidic flow-stretch chip for investigating blood vessel biomechanics. Lab Chip.

[41] Nabeshi H, Yoshikawa T, Matsuyama K, Nakazato Y, Matsuo K, Arimori A, Isobe M, Tochigi S, Kondoh S, Hirai T, Akase T, Yamashita T, Yamashita K, Yoshida T, Nagano K, Abe Y, Yoshioka Y, Kamada H, Imazawa T, Itoh N, Nakagawa S, Mayumi T, Tsunoda S, Tsutsumi Y (2011). Systemic distribution, nuclear entry and cytotoxicity of amorphous nanosilica following topical application. Biomaterials.

[42] Rouse JG, Haslauer CM, Loboa EG, Monteiro-Riviere NA (2008). Cyclic tensile strain increases interactions between human epidermal keratinocytes and quantum dot nanoparticles. Toxicol In Vitro.

[43] Parry SN, Ellis N, Li Z, Maitz P, Witting PK (2008). Myoglobin induces oxidative stress and decreases endocytosis and monolayer permissiveness in cultured kidney epithelial cells without affecting viability. Kidney Blood Press Res.

[44] Cheng J, Vieira A (2006). Oxidative stress disrupts internalization and endocytic trafficking of transferrin in a human malignant keratinocyte line. Cell Biochem Biophys.

[45] Sheetz MP (2001). Cell control by membrane-cytoskeleton adhesion. Nat Rev Mol Cell Biol.

[46] Cheng T (2001). Reactive oxygen species mediate cyclic strain-induced endothelin-1 gene expression via Ras/Raf/extracellular signal-regulated kinase pathway in endothelial cells. J Mol Cell Cardiol.

[47] Carosi JA, McIntire LV, Eskin SG (1994). Modulation of secretion of vasoactive materials from human and bovine endothelial cells by cyclic strain. Biotechnol Bioeng.

[48] Macarthur H, Warner TD, Wood EG, Corder R, Vane JR (1994). Endothelin-1 release from endothelial cells in culture is elevated both acutely and chronically by short periods of mechanical stretch. Biochem Biophys Res Commun.

[49] Wang DL, Wung BS, Peng YC, Wang JJ (1995). Mechanical strain increases endothelin-1 gene expression via protein kinase C pathway in human endothelial cells. J Cell Physiol.

[50] Sinha B, Köster D, Ruez R, Gonnord P, Bastiani M, Abankwa D, Stan RV, Butler-Browne G, Vedie B, Johannes L, Morone N, Parton RG, Raposo G, Sens P, Lamaze C, Nassoy P (2011). Cells respond to mechanical stress by rapid disassembly of caveolae. Cell.

[51] Glebov OO, Bright NA, Nichols BJ (2006). Flotillin-1 defines a clathrin-independent endocytic pathway in mammalian cells. Nat Cell Biol.

[52] Wong AJ, Pollard TD, Herman IM (1983). Actin filament stress fibers in vascular endothelial cells *in vivo*. Science.

[53] White GE, Gimbrone MA, Fujiwara K (1983). Factors influencing the expression of stress fibers in vascular endothelial cells *in situ*. J Cell Biol.

[54] Ingber DE (1997). Tensegrity: the architectural basis of cellular mechanotransduction. Annu Rev Physiol.

[55] Dai J, Sheetz MP (1995). Regulation of endocytosis, exocytosis, and shape by membrane tension. Cold Spring Harb Symp Quant Biol.

[56] Cho EC, Zhang Q, Xia Y (2011). The effect of sedimentation and diffusion on cellular uptake of gold nanoparticles. Nat Nanotechnol.

[57] Huh D, Matthews BD, Mammoto A, Montoya-Zavala M, Hsin HY, Ingber DE (2010). Reconstituting organ-level lung functions on a chip. Science.

[58] Peters K, Schmidt H, Unger RE, Otto M, Kamp G, Kirkpatrick CJ (2002). Software-supported image quantification of angiogenesis in an *in vitro* culture system: application to studies of biocompatibility. Biomaterials.

[59] Jaffe EA, Nachman RL, Becker CG, Minick CR (1973). Culture of human endothelial cells derived from umbilical veins. Identification by morphologic and immunologic criteria. J Clin Invest.

[60] Krump-Konvalinkova V, Bittinger F, Unger RE, Peters K, Lehr HA, Kirkpatrick CJ (2001). Generation of human pulmonary microvascular endothelial cell lines. Lab Invest.

