# Supplementary figures and images for: In vitro investigation of silica nanoparticle uptake into human endothelial cells under physiological cyclic stretch
Source: Part Fibre Toxicol. 2014 Dec 24;11:68. doi: 10.1186/s12989-014-0068-y (PMC4318365; doi:10.1186/s12989-014-0068-y)

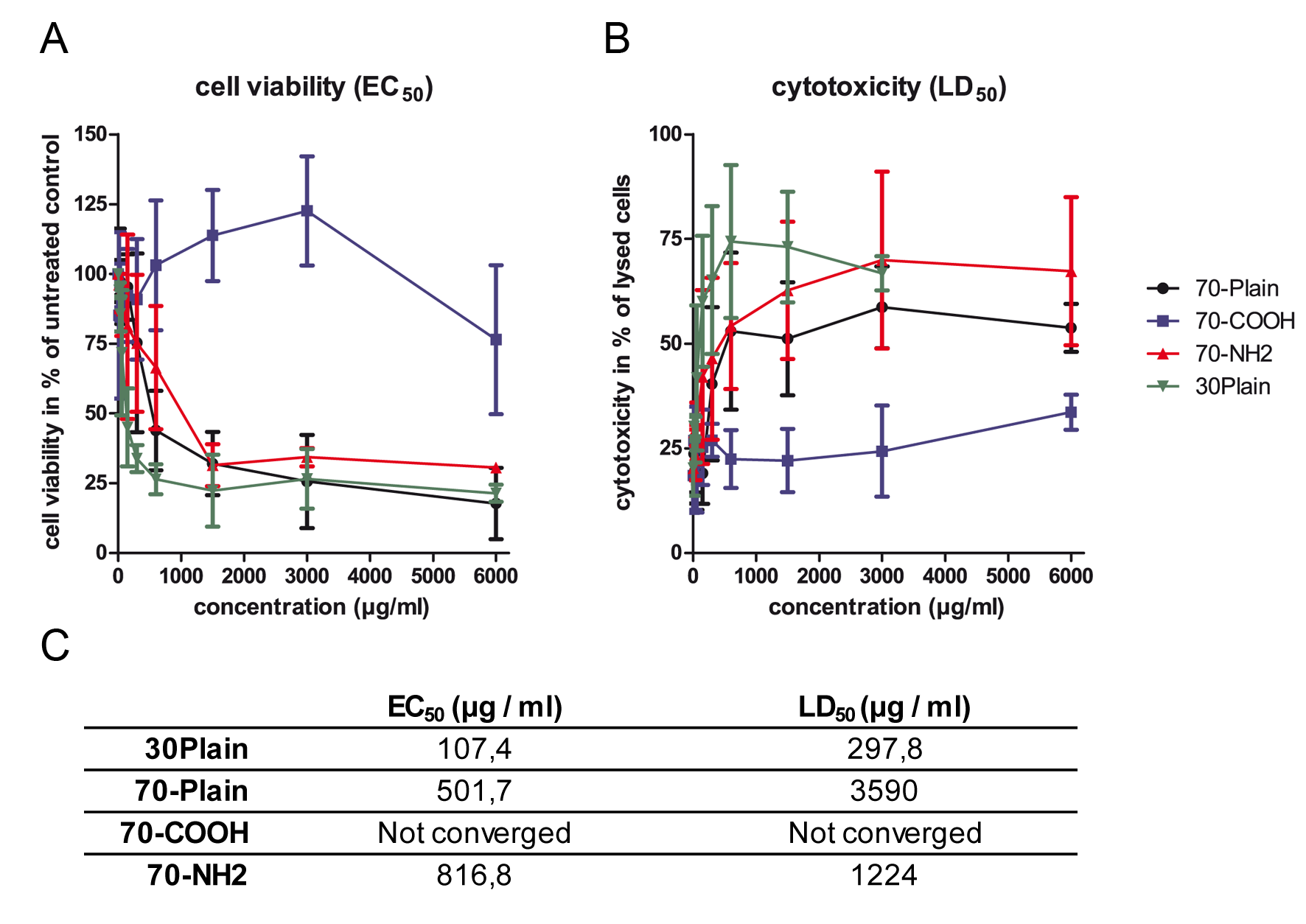

Supplement: Additional file 1: — Determination of EC 50 and LD 50 of various aSNPs in Huvec. (A) HUVEC were treated with various nanoparticles in a concentration range of 0 to 6000 μg/ml for 24 hours and EC50 was determined by MTS assay. Untreated cells were set to 100% cell viability (B) The supernatant of the cells was examined for the amount of released LDH to determine the LD50. Lysed cells were used as positive control and set to 100%. (C) Summary of EC50 and LD50 for the various aSNPs. (donors ≥3). [file 12989_2014_68_MOESM1_ESM.tiff]

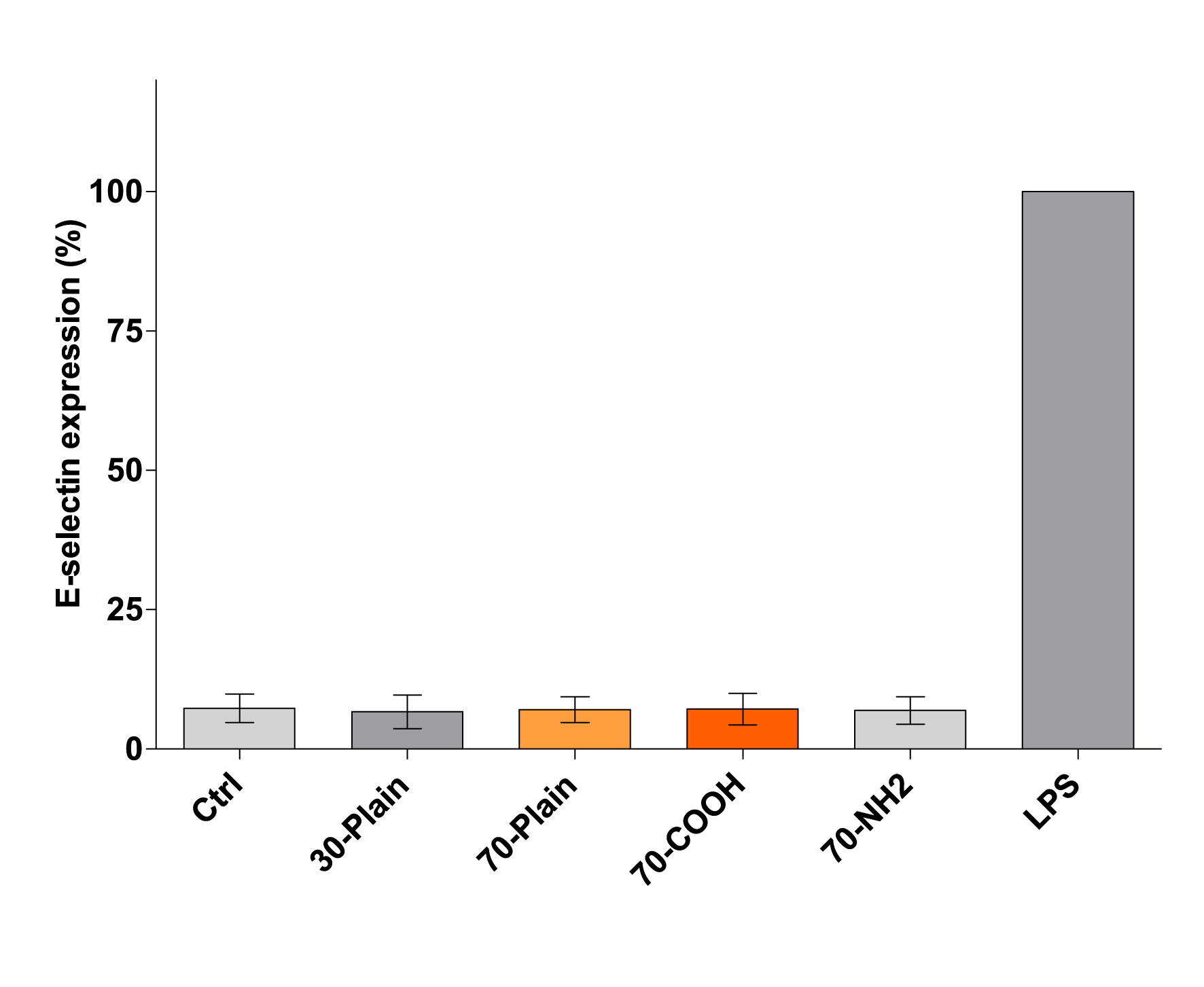

Supplement: Additional file 2: — E-Selectin expression of Huvecs to determine endotoxin contamination of the nanoparticles. HUVEC were grown on 96-well plates and treated with 60 μg/ml 30 nm-plain or 150 μg/ml 70 nm aSNPs for 4 hours. Cells were washed, fixed and E-selectin expression was determined by CAM-EIA as described by Unger et al. 2014 (see references). 1 μg/ml lipopolysaccharide was used as positive control and set to 100% E-selectin expression while untreated cells were used as negative control. (2 donors in triplicate). [file 12989_2014_68_MOESM2_ESM.tiff]

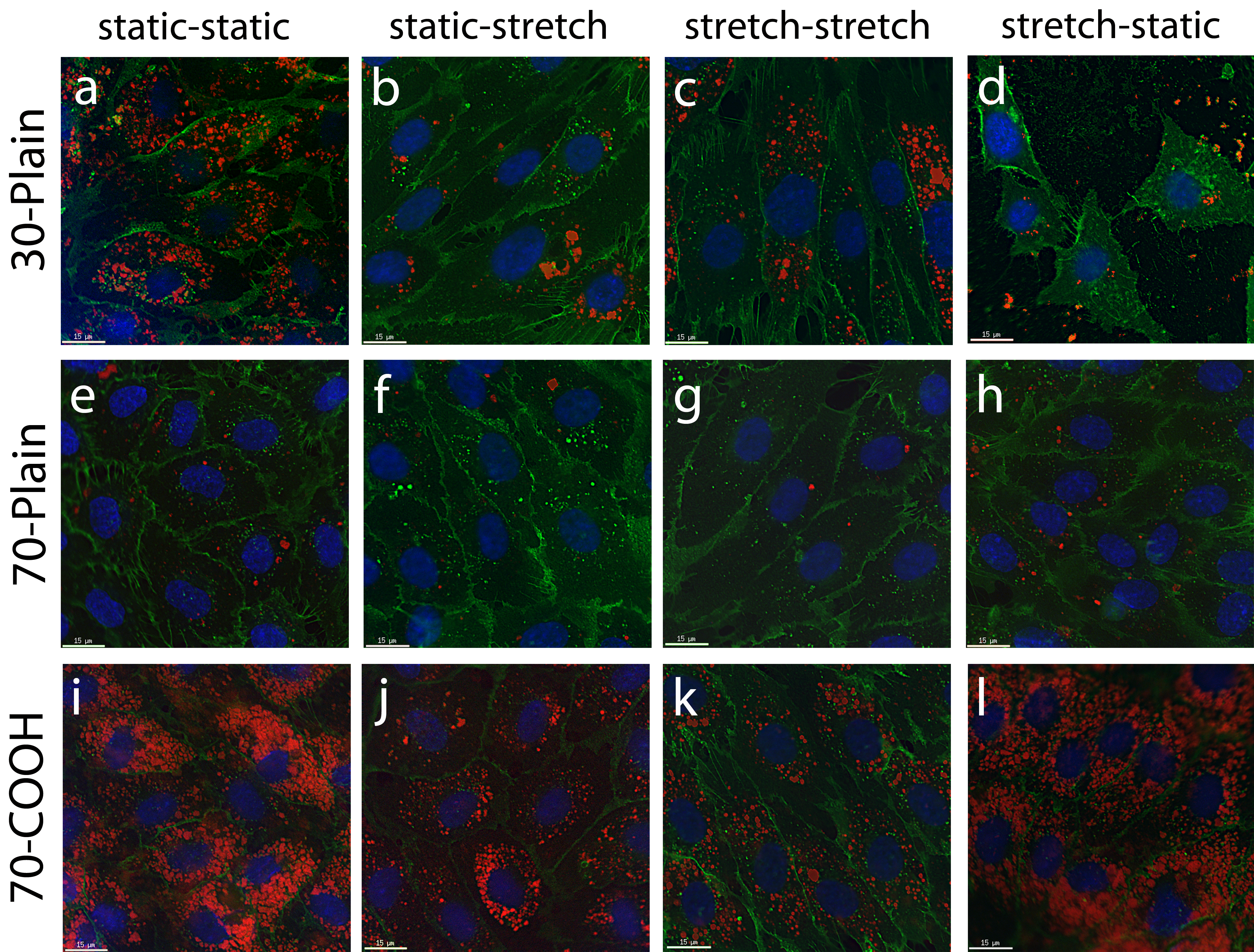

Supplement: Additional file 3: — Uptake of various aSNPs into HUVEC under static, stretch and mixed culture conditions. HUVEC cultivated on flexible membranes under static (a + b) and stretch (c + d) conditions were treated with silica nanoparticles under static (a + d) or stretch (b + c) conditions for 24 hours. Cells were extensively washed, fixed and stained (CD31 (green)). Cell nuclei were counterstained with Hoechst dye (blue). Scale bar: 15 μm. [file 12989_2014_68_MOESM3_ESM.png]

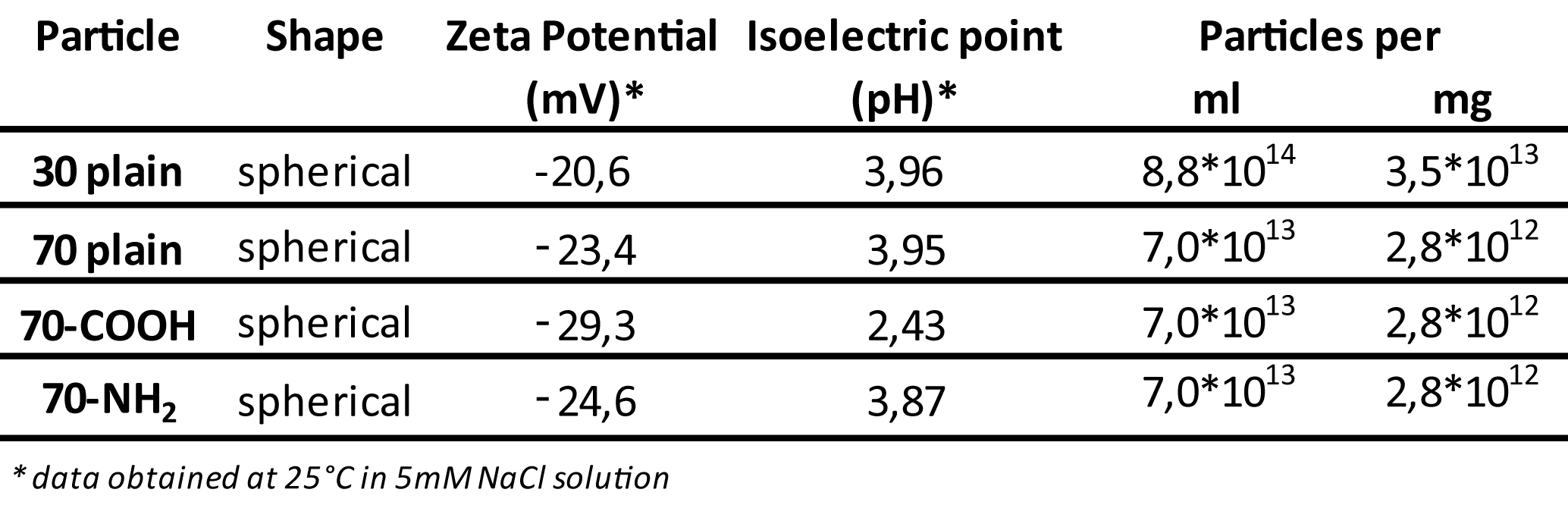

Supplement: Additional file 4: — Nanoparticle characteristics provided by the manufacturer ( www.micromod.de ). [file 12989_2014_68_MOESM4_ESM.tiff]
